# Predicting dog tracheal diameter and length: A tool for improved intubation

**DOI:** 10.5455/javar.2024.k780

**Published:** 2024-06-08

**Authors:** Maneenooch Khiao-in, Suppada Kananub, Tepyuda Sritrakul, Pattarawadee Thamsatit, Sirin Theerawatanasirikul, Naparee Srisowanna

**Affiliations:** 1 Department of Anatomy, Faculty of Veterinary Medicine, Kasetsart University, Bangkok, Thailand; 2 Department of Veterinary Public Health, Faculty of Veterinary Medicine, Kasetsart University, Nakornpathom, Thailand; 3 Critical Care Unit, Veterinary Teaching Hospital, Faculty of Veterinary Medicine, Kasetsart University, Bangkok, Thailand

**Keywords:** Endotracheal tube, dogs, formula, tracheal diameter, tracheal length

## Abstract

**Objective:**

This study aims to develop formulas for estimating tracheal diameter and length in dogs using easily measurable anatomical parameters.

**Materials and Methods:**

The samples consisted of 20 dogs of various breeds, comprising 10 males and 10 females, sourced from cadavers. The measured parameters included occipital tuberosity to tail base (OT), eye angle to ear tragus, nose to ear tragus, inner vertical diameters (IVD), and tracheal length (TL). The study conducted correlation and linear regression analyses, and subsequently, the formulated models underwent validation using 16 live dogs. The results were compared to radiographic measurements.

**Results:**

Linear regression recommended formulas based on OT, resulting in IVD (mm) = 0.203 × OT – 3.724 (*r^2^ =* 0.608, *p* < 0.001) and TL (cm) = 0.346 × OT–3.773 (*r^2^ =* 0.837, *p* < 0.001). The predicted tracheal diameter and length from formulas were slightly smaller than radiographic measurements (IVD = 2.76 ± 1.85 mm, *p* < 0.0001 and TL = 2.07 ± 1.81 cm, *p* < 0.0001).

**Conclusion:**

These formulas offer a practical way to estimate tracheal dimensions in live dogs, facilitating the selection of suitable endotracheal tube sizes and insertion depth. Further studies with larger sample sizes and consistent measurement methods can enhance the accuracy of these findings.

## Introduction

Endotracheal tube (ETT) intubation is a crucial procedure performed across various medical contexts, including general anesthesia, critical care, and emergencies. Its aims encompass maintaining airway clarity, efficient oxygen, anesthetic delivery, and safeguarding against aspiration and foreign material ingress [1,2]. Employing an ETT possessing the maximum diameter capable of passing easily through the narrowest part of the airway is recommended [3]. This minimizes airway resistance, easing breathing effort [4,5]. However, overly large ETT can cause trauma or intubation failure, while excessively small ETT may lead to gas leakage and room contamination. Narrower ETT increases airway resistance, intensifying breathing work [6,7]. Proper length involves positioning the ETT connector external to the incisors, and its bottom midway between the larynx and thoracic inlet [3–5]. Incorrect placement can yield severe consequences. Over-intubation into the right mainstem bronchus increases the risks of hypoventilation, pneumothorax, and atelectasis. Conversely, under-insertion may incorrectly position the cuff over the vocal cords, causing trauma or accidental extubation [8,9].

Veterinarians often face challenges in selecting the appropriate ETT size for intubation in dogs because of the anatomical diversity and size differences among breeds [3,10]. Despite various suggested methods, no standard technique exists for accurate ETT size selection [7,10]. Common methods include predicting ETT size based on lean body weight [4] or externally palpating the trachea [7,11]. However, both methods present limitations: significant differences in weight among various body condition scores [3,10], the external and internal diameters of tracheal tubes differ significantly, and the diameter of the tracheal lumen narrows upon entering the thoracic cavity [12].

Thoracic radiography, fluoroscopy, bronchoscopy, and ultrasonography have been used to assess tracheal diameter in dogs. Nonetheless, these techniques are constrained by equipment availability, time consumption, and the expertise required [13]. The formula is a method used to estimate endotracheal size and depth. However, there are only a few specific formulas developed specifically for dog applications, and only a few studies are available [10,14]. In contrast, in human medicine, especially in pediatrics, multiple formulas have been proposed to predict optimal ETT size and insertion depth [15,16]. Notably, studies in dogs primarily focused on predicting tube size rather than intubation depth [10,14,17]. Studies investigating the relationship between ETT size or tracheal diameter and body parameters mainly focused on assessing the dimensions of the nose, philtrum, and digital pad. The studies also considered body mass (kg), body size (cm), and age as variables. Avki et al. [14] proposed equations for Dalmatian puppy endotracheal sizing using body mass and vertical length of the fourth digital pad. Unfortunately, the formulas were not reported as tested results in other groups of dogs. Another recent study by Haider et al. [10] studied the internal tracheal diameter correlation with body mass (kg), body size (cm), fourth digital pad width, the height of the left forelimb, width, and height of metacarpal and carpal pad, length of the philtrum, and the distance between the philtrum and the lateral edge of the nares. The strongest correlation was with body mass. The validation result was strongly accurate for mesocephalic and dolichocephalic dogs. Despite consistent results, variations in body condition scores may impact estimation accuracy. Tong and Pang [17] explored several published formulas and examined the relation between body measurements and the cube root (^3^√) of body mass for selecting ETTs.

Therefore, the prediction of the actual diameter and depth of the ETT is important and should be individualized. This study aimed to develop a formula to help the practitioner select the appropriate ETT size in dogs based on body parameters that are not relevant to body condition scores and are easily measured directly by dogs.

## Materials and Methods

### Ethical approval

This study was conducted at the Department of Anatomy, Faculty of Veterinary Medicine, Kasetsart University, with approval from the Kasetsart University institutional animal care and use committee (ID# ACKU66-VET-061). A sample of 20 variety-breed dog cadavers, evenly split between males and females, was used. These cadavers were sourced from undergraduate student dissection classes.

### Measurement

**Figure 1.**
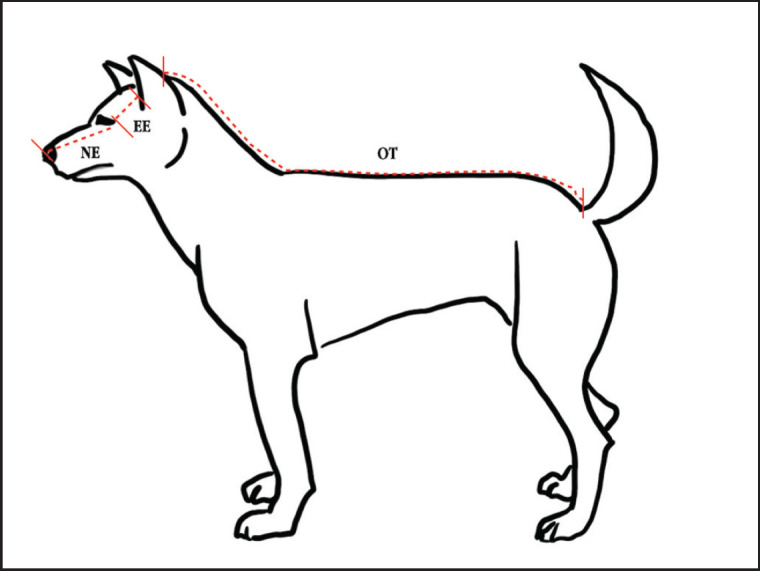
Anatomical positions for measuring three distinct body parameters: 1) OT, which is the distance from the occipital tuberosity to the base of the tail; 2) EE, representing the distance from the angle of the eye to the tragus of the ear; and 3) NE, indicating the distance from the nose to the tragus of the ear.

**Figure 2.**
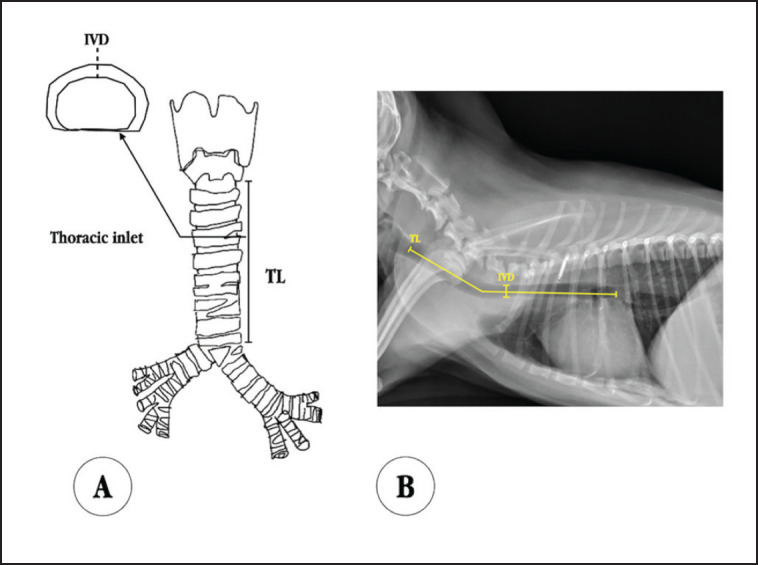
Tracheal measurement. A: Tracheal diameter was measured as IVD at the position of the first intrathoracic tracheal ring. TL was measured from the cranial margin of the initial tracheal ring to the point of bifurcation. B: The IVD was measured from the radiograph by drawing a vertical line from the dorsal to ventral aspect of the trachea, at the position anterior to the first rib. The TL was measured from the cranial margin of the initial tracheal ring (observe cricoid cartilage at second cervical vertebrae) to the point of bifurcation.

Body distances, tracheal length (TL), and tracheal diameter were measured utilizing specific tools: a measuring band, metal ruler, and digital Vernier calipers (Metric and Inch Series 500-196-30, Mitutoyo, Japan), respectively. These body distances included three distinct measurements: 1) occipital tuberosity to tail base (OT), 2) eye angle to ear tragus (EE), and 3) nose to ear tragus (NE) (Fig. 1). Tracheas were excised from the cadavers, and measurements of inner vertical diameters (IVD), were taken at the thoracic inlet tracheal ring. TL was defined as the measurement from the cranial margin of the first tracheal ring to the point of bifurcation (Fig. 2A).

### Statistical analysis

Descriptive statistics for all parameters were presented as mean ± SD. The normality was tested by the Shapiro-Wilk. The comparison of the means was conducted with a *T*-test analysis. The relationships between the measured parameter data (OT, NE, and EE) and IVD or TL were analyzed by two-tailed Pearson correlation. The correlation was accepted as significant when* p* < 0.05 and regarded as a high *R*-square (*R*^2^) value. Subsequently, a linear regression analysis was performed using IVD and TL as dependent variables and correlating phenotypic parameters as independent variables.

In the validation step, 16 live dogs without airway disease histories were included. The diameter and length were determined radiographically. Diameter measurements were taken anterior to the first rib at the thoracic inlet position, while length measurements were taken along the tracheal line from behind the cricoid cartilage to the bifurcation (Fig. 2B). The *T*-test analysis was used to compare formula-based results with radiographic measurements.

Statistical analyses utilized SPSS Statistics Version 26 software (SPSS Inc., Chicago, IL), and graphs were generated using GraphPad Prism Version 9.5.1 (GraphPad Software, Boston, MA).

## Results

The average age of 20 dogs was 12 ± 4.75 years (1.5–18 years). The most common breeds were mixed breeds (*n =* 12), pugs (*n =* 2), Siberian Huskies (*n =* 2), Corgi (*n =* 1), French Bulldog (*n =* 1), Pit Bull (*n =* 1), and Poodle (*n =* 1), with no history of airway disease. All parameters are summarized in Table 1. Notably, all these measurements demonstrated a normal distribution, except for NE, which did not follow a normal distribution. Despite this, no significant differences were observed between the sexes in any of the measurements of IVD (*p* = 0.87), TL (*p* = 0.2), OT (*p* = 0.07), EE (*p* = 0.39), and NE (*p* = 0.26), as indicated by the *T*-test results.

The IVD and TL showed a very strong correlation between them (*r =* 0.800, *p* < 0.001). The OT was the body parameter that showed the best correlation to IVD when compared to the others. They had a moderate correlation (*r =* 0.780, *p* = 0.01). While the correlation between IVD and EE, NE was fair (*r =* 0.471, *p* = 0.05, and *r =* 0.573, *p* = 0.01, respectively), as shown in Table 2, applying the most correlated variables, linear regression suggests the predicted formula. This formula yielded an *R*^2^ value of 0.608 (*p* < 0.001). The diameter formula was as follows:

**Table 1.** Presents data for the IVD, TL, and the measurements of OT, EE, and NE in a sample of 20 dog cadavers. The data is presented as mean ± standard deviation (minimum to maximum).

Variables	All (*n =* 20)	Male (*n =* 10)	Female (*n =* 10)
IVD (mm)	9.33 ± 2.56 (5.37–13.88)	9.96 ± 2.46 (5.44–13.59)	8.70 ± 2.63 (5.37–13.88)
TL (cm)	18.48 ± 3.70 (11.5–25.50)	19.50 ± 4.03 (11.5–25.5)	17.45 ± 3.27 (11.5–22)
OT (cm)	64.30 ± 9.84 (42–81)	68.20 ± 9.73 (48–81)	60.40 ± 8.72 (42–69)
EE (cm)	7.52 ± 1.38 (5–10)	7.8 ± 1.57 (5–10)	7.25 ± 1.18 (5–9)
NE (cm)	16.7 ± 2.69 (10–21)	17.40 ± 2.87 (11–21)	16 ± 2.45 (10–18)

IVD (mm) = 0.203OT (cm) – 3.724

**Figure 3.**
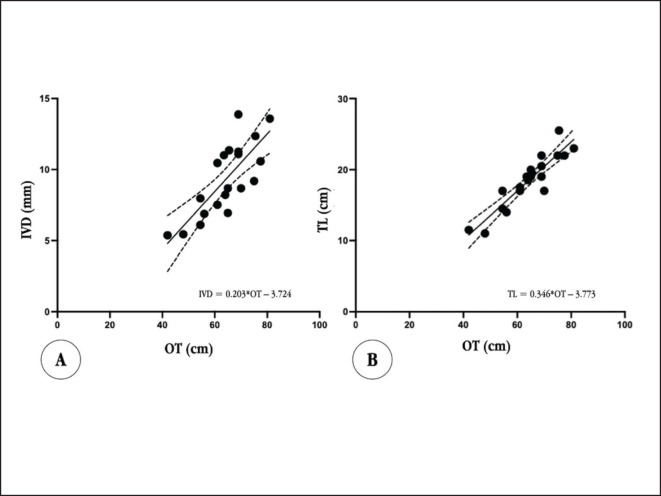
A: presents linear regression between OT and IVD B: presents linear regression between OT, and TL both with 95% confidence.

Furthermore, TL exhibited the highest correlation with OT, akin to IVD. The correlation coefficient of TL and OT was 0.915, with a *p ≤* 0.0001, indicating a very strong correlation. Conversely, the correlation between TL and EE was relatively moderate (*r =* 0.595, *p = *0.006). On the other hand, the correlation between TL and NE was strong (*r =* 0.864, *p* < 0.0001), as hown in Table 2. Despite the strong correlation shown between TL, OT, and NE variables, the linear regression analysis suggested a formula incorporating only OT. This equation yielded an *R*^2^ value of 0.837 (*p* < 0.001) (Fig. 3). The TL formula was as follows:

TL (cm) = 0.346OT (cm) - 3.773

**Figure 4.**
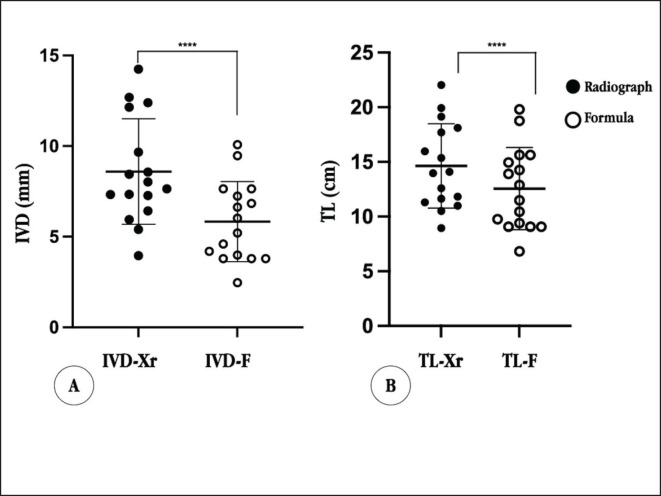
Presents scatter plots and mean ± SD comparisons of the results from the validation group. A: IVD B: TL Significant differences were observed within both groups (*p* ≤ 0.0001).

The formulas were validated on 16 live dogs (8 males and 8 females) without a respiratory disease history. The average ages of validated group dogs ranged from 2.2 to 15.3 years, averaging 11 ± 3.28 years. The sample consisted of mixed breeds (*n =* 3), Pomeranian (*n =* 3), Thai Bang Kaew (*n =* 1), Shih Tzu (*n =* 3), Chihuahua (*n =* 4), and Poodle (*n =* 2). The tracheal diameter and length were measured from radiographs, incorporating the OT variable in both the IVD and TL formulas. The predictive mean diameter was 5.83 ± 2.21 mm, while radiographic measurements yielded a mean diameter of 8.59 ± 2.91 mm. The difference in the mean values is 2.76 ± 1.85 mm (*p* < 0.0001). Similarly, the predictive mean TL was 12.56 ± 3.77 cm, while the radiographic mean length was 14.63 ± 3.85 cm. The difference in the mean values is 2.07 ± 1.81 cm (*p* < 0.0001). Notably, significant differences in mean values and standard deviations were found between the results from the predictive formula and the radiographic measurement. Remarkably, our tracheal diameter and length from formulas consistently trended smaller compared to the diameter and length from radiographic measurements (Fig. 4).

**Table 2.** Presents Pearson’s correlation coefficients among different parameters.

	TL	IVD	OT	EE	NE
TL	1	0.800****	0.915****	0.604**	0.868****
IVD		1	0.780****	0.471*	0.573**
OT			1	0.558*	0.871****
EE				1	0.731**
NE					1

* *p* ≤ 0.05,

** *p* ≤ 0.01,

*** *p* ≤ 0.001,

**** *p* ≤ 0.0001.

## Discussion

This study aimed to demonstrate the relationships between IVD, TL, and other measurable anatomical features of dogs. The goal was to develop accurate formulas for calculating tracheal diameter and length. These formulas are designed to help practitioners choose the right ETT size and insertion depth for dogs. In this study, the selection of the IVD to represent the tracheal diameter was based on its recognition as the narrowest dimension within the thoracic inlet region [12,18,19]. This approach also helps to avoid the erroneous use of the external diameter measurements obtained by palpating the trachea from the outside [7,11]. In this study, all measured parameters showed clear sex independence, demonstrating the formula’s applicability to both genders. Surprisingly, before this study, the existence of sex-independent tracheal dimensions in dogs had not been addressed. In studies involving human subjects, no significant correlations were found between tracheal diameter and gender [20]. However, it is worth highlighting that certain investigations in humans have pointed out variations based on height, which is a strong predictor of tracheal diameter, particularly differing between sexes [21–23].

The selected parameters are user-friendly for measurement and are not affected by body condition scores. This is important because body condition scores can affect the length of body parameters, which could lead to inaccurate estimates of tracheal diameter [3,10]. However, the body parameters from previous studies that exhibited a strong correlation with tracheal diameter, such as the vertical length of the fourth digit [14] and the width of the nose [17], were not included in these studies, even though they are also not affected by body condition scores. The reason is that the formula with the vertical length of the fourth digit did not undergo a validation step, and the result from the formula with the width of the nose was not precise [14,17]. The other parameters related to the dimensions of the nose, philtrum, and digital pad did not show a correlation with tracheal diameter and length [17].

The results of this study revealed significant correlations between IVD and TL itself. Noticeably, both IVD and TL exhibited strong correlations with OT. These correlations are consistent with previous reports in humans, where studies have indicated a strong correlation between tracheal diameter and length and the height of the entire body [24,25].

The linear regression suggested both formulas incorporating only OT. The advantage of this approach is its practicality for practitioners, who can measure only one body length for calculation. However, during the validation test, it was observed that both the tracheal diameter and length formulas yielded smaller results when compared to the diameter and length measured from radiographs of live dogs. This inaccuracy may arise from the effects of tracheal diameter and length in cadavers and radiographs. Tracheal diameter and length in cadavers, subjected to a fixative solution (formalin-based) for a year, might experience shrinkage. This shrinkage phenomenon is well documented in various studies involving hollow organs, such as the circumference of the heart valve [26]. To date, there have been no direct studies on tracheal shrinkage. However, studies on head and neck or esophagus tissues have demonstrated shrinkage ranging from 4.4% to 10% after just 48 h of formalin fixation [27]. Furthermore, significant shrinkage has been observed in the mucosa attached to cartilage and cartilage itself after being fixed in 10% formalin for 24 h [28]. In radiographs, the tracheal lumen exhibits dynamic movement, responding to the fluctuating internal pressure of the respiratory cycle (inspiration and expiration), leading to changes in its dimensions [29,30]. It is conceivable that this phenomenon may have influenced the data in the validation group.

The study has some limitations. The formulas were established using a relatively small number of dogs, which exhibited variations in breeds, ages, and sizes. Additionally, differences in measurement methods may introduce errors in the predictive formulas. Consequently, further research should involve a larger and more carefully categorized sample, considering variations in breeds, ages, and sizes. It is recommended to utilize diameter and length measurements from radiographs for both groups.

## Conclusion

In conclusion, this study contributes valuable insights into the relationships between tracheal dimensions and anatomical parameters in dogs, offering potential applications for ETT size selection and depth of insertion. Nevertheless, it is essential to acknowledge the limitations, including the relatively small sample size and the impact of differences in tracheal diameter and length measurements between cadavers and radiographs. To refine these findings, further research with a large number of live dog samples, along with consistent measurement methods for tracheal dimensions, would help improve the accuracy of the predictive formulas. Ultimately, an improved understanding and application of tracheal measurements have the potential to greatly benefit veterinary practitioners and enhance the well-being of canine patients.

## List of abbreviations

EE, eye angle to ear tragus; ETT, Endotracheal tube; IVD, inner vertical diameters; NE, nose to ear tragus; OT, occipital tuberosity to tail base; TL, tracheal length; mm, millimeter; cm, centimeter; kg, kilogram.
